# First Clinical Nutrition Outpatient Consultation: A Review of Basic Principles in Nutritional Care of Adults with Hematologic Disease

**DOI:** 10.1155/2023/9303798

**Published:** 2023-09-21

**Authors:** Julia da Silva Goncalves dos Santos, Barbara de Farias Meirelles, Isabela de Souza da Costa Brum, Mariana Zanchetta, Bruna Xerem, Lucas Braga, Marcia Haiut, Renata Lanziani, Taha Hussein Musa, Karen Cordovil

**Affiliations:** ^1^Institute of Hematology Arthur Siqueira Cavalcanti (Hemorio), Rio de Janeiro, RJ, Brazil; ^2^Oswaldo Cruz Foundation (Fiocruz), Rio de Janeiro, RJ, Brazil; ^3^Biomedical Research Institute, Darfur University College, Nyala, Sudan

## Abstract

**Methods:**

A bibliographic survey was carried out between 2020 and 2022 using two databases: PubMed/MEDLINE and Scientific Electronic Library Online (SciELO) and the information source Academic Google, irrespective of language or geography.

**Results:**

In the first nutrition consultation (FNC), there should be an investigative direction focused on nutritional interventions in the short, medium, and long term. The record in the patient's medical record is relevant for carrying out the consultation, according to the recommendations of the normative councils of medicine and nutrition. The main steps to be followed are the investigation of the presence of food allergies and intolerances; the drugs/nutritional supplements in use; changes in the digestive tract; the presence or absence of picamalacia; and socioeconomic and lifestyle data. In addition, it is necessary to carry out laboratory evaluations, semiological assessment, anthropometric assessment, and assessment of food consumption. In the end, the nutritional approach should be composed of calculation of energy and macronutrient and micronutrient needs, intervention in nutritional status deviations, nutritional guidelines, and nutritional therapeutic planning of return, focusing on adherence to treatment.

**Conclusion:**

The first nutrition consultation may represent investigative steps that help the clinical nutritionist in the management, allowing a longitudinal and specific nutritional therapeutic planning for patients assisted in large reference centers for hematological disease.

## 1. Introduction

Pathophysiological changes in the blood system have led to the emergence of several types of hematological disorders in millions of people annually worldwide, which generate hematological diseases (HD) characterized by functional impairment in the production of blood cells, such as erythrocytes (red blood cells), leukocytes (white blood cells), and platelets (thrombocytes) [1].

HD can generally be classified as proliferative and infiltrative (PI), anemias (A), and alterations in coagulation (AC) (Table 1), and among them, myeloproliferative diseases, hemoglobinopathies, and hemophilia deserve to be highlighted [2, 3].

Polycythemia vera (PV) is a type of PI that has a prevalence of 44–57 per 100,000 people in the United States [8]. Multiple myeloma (MM), another type of IP, has an estimated incidence in Europe of 4.5–6.0 per 100,000 cases/year [9]. Among anemias, the worldwide incidence of sickle cell disease, glucose-6-phosphate dehydrogenase (G6PD) deficiencies, and thalassemia was estimated at 0.61, 7.54, and 0.14 million cases/year in 2017, respectively. G6PD deficiency has the highest epidemiological burden worldwide, followed by other hemoglobinopathies [10]. Hemophilia A, a type of CA, stands out with a prevalence of 0.95 cases per 10,000 inhabitants in Brazil [11].

Clinical treatment of HD is usually performed by a hematologist, and/or hemotherapist, and other professionals who make up the multidisciplinary team. Nutritional care is important in acute or chronic HD, especially in the control of metabolic and gastrointestinal disorders that will affect the nutritional status of the individual [12].

Nutritional care usually focuses on careful clinical evaluation, which includes physical examination and dietary history, considering the determination of the patient's functional capacity, disorders present in the digestive tract, and risk factors for complications secondary to pre-established diseases [12, 13]. Nutritional assistance in HD must always be centered on the person and considered the same as a complex being that cannot be divided into parts [14–16].

Therefore, the outpatient follow-up of patients with HD demands specialization from the clinical nutritionist, especially regarding issues related to nutritional aspects and eating habits, which can be directly and/or indirectly associated with the individual's underlying disease [17–21].

It is at the first nutrition consultation (FNC) that there should be an investigative approach focused on short-, medium-, and long-term nutritional diagnoses and interventions, considering the food and nutrition needs of individuals with HD [12]. Thus, we have the initial proposal to review or carry out a narrative review of the basic principles in the first-time follow-up of outpatients in reference centers for hematological disease. In addition, we propose strategies and recommendations that can guide the nutrition professionals in the nutritional care of adults with hematological disease.

## 2. Methods

A bibliographic survey was carried out between the years 2020 and 2022 using two databases: PubMed/MEDLINE and Scientific Electronic Library Online (SciELO) and the information source Academic Google, irrespective of language or geography. For the search, the Boolean operator “OR” was used in the groups of keywords: “hematological diseases”; “patient's chart”; food allergy”; “food intolerance”; “drug and nutrient interaction”; “gastrointestinal tract changes”; pica; lifestyle; “laboratory tests”; semiology; anthropometry; “food consumption”; and “energy needs”. After that, the Boolean operator “AND” was used to obtain the intersection between the keyword hematological diseases and the first group of keywords mentioned above.

## 3. FNC Implementation

The construction of normative steps to be implemented and followed in the FNC can be organized collectively by the technical team of the Nutrition and Dietetics service of the hospital, health unit, or reference center for HD patients.

For the construction of the FNC, technical and scientific manuals can be used, considering the nutritional aspects aimed at the nutritional monitoring of adult patients with the hematological disease and respecting the normative aspects of the institution [12]. In addition, participant observation can be a qualitative method to be used by the nutrition team in the construction of the FCN, as it is an ethnographic observation approach in which the observer actively participates in data collection activities, requiring the researcher's ability to adapt to a given situation [22].

### 3.1. Basic Procedures for Outpatient Consultation

Hospital accreditation is a systematic evaluation practice, to guarantee within established minimum standards the quality of care [23]. The accreditation process has an impact on the consolidation of a culture of quality and patient safety and leads to significant organizational changes, permeated by the standardization of care processes and the establishment of performance management systems [24, 25].

In Brazil, the accreditation methodologies that most certify health services are those applied by the Joint Commission International (JCI) and the National Accreditation Organization (NAO), which adopted the JCI and Canadian Accreditation models to the Brazilian context [26].

JCI, represented in the Brazilian scenario by the Brazilian Accreditation Consortium (BAC), is an organization responsible for promoting and ensuring the continuous improvement of the quality of care in health institutions, through international consensus standards, international safety goals, and patient and monitoring assistance with indicators. The patient's medical record, for example, is a fundamental document in quality control and hospital accreditation [27, 28].

In Brazil, the Federal Council of Medicine, through Resolution 1638/2002, defines the patient's medical record as a single document consisting of a set of information, signs, and recorded images, generated from facts, events, and situations about the patient's health and the assistance provided to him, of a legal, confidential, and scientific nature, which enables the communication between members of the multidisciplinary team and the continuity of the assistance provided to the individual [29].

According to this resolution, the doctor must record the following data in the medical record: patient identification (full name, social name, date of birth, sex, mother's name, place of birth, and full address), anamnesis, physical examination, complementary exams requested and their respective results, diagnostic hypotheses, definitive diagnosis, and treatment performed, in addition to the daily evolution of the patient, with date and time, the discrimination of all the procedures to which he was submitted, and the identification of the professionals who performed them [29].

In addition to the above, outpatient nutrition consultations must include the following information: the time record (date in day, month, and year and the time in hours and minutes of the service), age of the individual, type of consultation (first time or return), underlying hematologic disease as the main diagnosis, associated comorbidities, nutritional diagnosis, dietary prescription, and the evolution of nutritional status all in the form of a header [12, 30].

It is important to emphasize that the medical record is the property of the patient and that the health professional must guarantee the patient the freedom of access to the data of his clinical history [31]. When properly performed, the medical record can contribute to teaching, research, the preparation of censuses, proposals for public health care, and the assessment of the quality of care provided [32].

The first step is to record information relevant to the nutrition consultation in the medical record in the form of a header. This item is important as it allows the identification of risks, diagnosis, treatment, and monitoring of the evolutionary state of health care, promoting the longitudinal continuity of care [26, 33].

### 3.2. Presence of Food Allergies and Intolerances

According to the Brazilian Consensus on Food Allergy [34], adverse food reactions are defined as any abnormal reaction to the ingestion of food or food additives and can be classified into toxic and nontoxic. Toxic reactions are those more related to the substance ingested or the pharmacological properties of certain substances present in food.

Nontoxic reactions are those that depend on individual susceptibility and are classified as immune-mediated (food hypersensitivity or food allergy) or non-immune-mediated (food intolerance). Hypersensitivity or allergy is an adverse reaction dependent on immunological mechanisms, whether immunoglobulin E (IgE) mediated or not, which can determine several clinical syndromes [34]. In FNC, the nutritionist should ask patients if they have food allergies and/or intolerances according to Table 2.

### 3.3. Medications in Use

Most drugs are administered orally, making drug-nutrient interactions (DNI) much easier. Therefore, reduction or exacerbation of the drug's therapeutic effect is possible, as well as a modification in the nutrient's bioavailability, with clinical implications on pharmacological therapeutic efficacy and nutritional status. Patients should be individually evaluated for the effect of food on medication action and the possible effect of medication use on nutritional status [35].

In addition to medications, many patients use food supplements, whether medicated or not, defined as oral products, presented in pharmaceutical forms, intended to supplement the diet of healthy individuals with nutrients, bioactive substances, enzymes, and probiotics, alone or in combination [36]. Phytotherapies are products obtained from a medicinal plant, or its derivatives, except for isolated substances, with prophylactic, curative, or palliative purposes [37]. The nutritionist, within the scope of his/her competencies, may prescribe food supplements, as well as foods for special and herbal purposes, by current legislation, when necessary [38].

Thus, the nutritionist must describe all the drugs used by the patient and their respective commercial name, as well as the food and herbal supplements, always questioning and recording in the medical record the name of the drug, the commercial name, dosage, and frequency of use. The DNI may be carried out in subsequent consultations.

### 3.4. Gastrointestinal Tract Changes

Abnormal symptoms related to gastrointestinal function can occur from the time food is swallowed until the time stool is expelled from the body [39]. Dysphagia, heartburn, bloating, abdominal pain, and changes in bowel habits are very common in the general population, and these changes are influenced by several factors, of a nonmodifiable nature, related to genetic inheritance, age, and sex and, of a modifiable nature, related to the environmental context, such as poor diet, smoking, and physical inactivity [39, 40].

Some hematological diseases and their respective treatments can cause changes in the gastrointestinal tract with an impact on the quality of life of affected individuals [41–43]. Antineoplastic chemotherapy and radiotherapy, for example, can cause gastrointestinal toxicity effects such as anorexia, nausea, vomiting, mucositis, diarrhea, or constipation, thus compromising the nutritional status and quality of life of people with cancer [44, 45].

In sickle cell disease (SCD), characterized as a structural type hemoglobinopathy, many people may have hypophagia and anorexia [46, 47]. In a cross-sectional study of two hundred and one people with SCD, most participants with the HbSS genotype had more symptoms of anorexia when compared to people with the HbSC genotype [48]. In addition, hydroxyurea, one of the drugs used in the treatment of SCD, could cause gastrointestinal toxicity, such as nausea and anorexia [49].

Thus, in the FNC, the nutritionist must question and record in the medical record whether patients have had some changes in the digestive tract (Table 3), considering the intensity and frequency of symptoms, to assess possible complications that could have a significant impact on the nutritional status of people with HD.

### 3.5. Picamalacia

Picamalacia or Pica can be defined as an eating disorder characterized by the persistent ingestion of inappropriate substances with little or no nutritional value, or of edible substances, but not in their usual form [65]. It includes geophagia (land consumption), amylophagy (raw starch consumption), pagophagia (ice consumption), and other forms of nonfood consumption [66]. Pica is common among vulnerable populations, especially pregnant women and children, and has been linked to both positive and negative health effects [67, 68]. Ingestion of nonfood substances causes complications that can be harmful or even fatal, which include electrolyte imbalance, metabolic disturbances, tooth damage, intestinal obstruction, or perforation, choking, infection, and poisoning [69, 70].

In an outpatient clinical practice of nutrition in hematology, it is common to observe a higher frequency of pica in individuals with SCD [12]. Some studies have shown that patients with SCD exhibit pica behaviors at some point in their lives, and the consumption of items is described as uncommon, persistent, and compulsive in nature [69, 71]. Although the etiology of pica in these patients is still not well understood, it is believed that there is a relationship with the deficiency of some micronutrients, such as iron and zinc [72–74].

A cohort study observed that individuals with SCD and pica had significantly lower hemoglobin levels when compared to those without pica [71]. Furthermore, in iron-deficient patients, the most consumed item during pica behavior is ice. Although ice is not a source of iron, it is hypothesized that chewing ice causes a vasoconstrictor response that can result in increased brain perfusion and may lead to increased alertness and processing speed. Individuals with iron deficiency often experience sluggishness because of effects resulting in decreased oxygen delivery to the brain [75, 76].

The diagnosis of pica is made if the ingestion of nonnutritive substances persists for at least 1 month, the eating behavior is not part of a culturally sanctioned practice, and the behavior is not simply a symptom of another mental disorder [71, 77]. Due to the severity of these complications, the therapeutic approach should be centered on reducing pica behavior to zero occurrences [69].

It is important that the provider performs routine screening for dysfunctional eating patterns and pica in patients with hematologic disease, especially those with sickle cell disease [78]. Thus, the nutritionist should ask the patient for episodes of pica according to Table 2.

### 3.6. Socioeconomic Conditions and Lifestyle

The social determinants of health can be defined as all social, economic, political, environmental, and cultural circumstances in which people are born, grow, live, work, and age, also including the local health system [79]. All these conditions influence people's health status, affecting them under different mechanisms, from physical-material aspects related to income inequalities, as well as psychosocial and social capital factors [80].

Lifestyle can be defined as the set of habits and customs that are influenced, modified, encouraged, or suppressed by the socialization process. These habits and customs range from the practice of physical activity to dietary habits such as the use of substances such as alcohol and tobacco [81].

Currently, chronic diseases, such as cardiovascular diseases and cancer, are considered preventable health problems. It is widely recognized that unhealthy lifestyles are major risk factors for many chronic diseases and premature death [82, 83]. Thus, lifestyle can reflect on patterns of behavior and daily habits, which, associated with living conditions, can have an important impact on a person's health.

Understanding that socioeconomic and lifestyle factors can play a central role in determining people's health, the nutritionist must have a broader view of these issues since they are factors that influence health and nutritional care. In this way, we must question some important factors according to Table 2.

### 3.7. Laboratory Evaluation

Laboratory tests can be considered cost-effective tools to obtain information about the patient's health status. They provide information that will be used for diagnosis, prognosis, prevention, and establishment of risks of various pathologies, as well as monitoring pre-established pathologies, to restore the quality of life of individuals [84, 85].

Laboratory evaluation helps in the clinical practice of the nutritionist, as we identify early changes in nutritional status. In addition, it is necessary to evaluate the nutritional evolution of the patient, as well as to provide a more accurate nutritional diagnosis, giving a fundamental predictive role for the effectiveness of diet therapy [86].

In practice, when working in a unit specialized in hematology, hematologists routinely request tests that can be used by the nutritionist to assess nutritional status and energy expenditure, predict inadequacies, and propose dietary interventions. Although patients already have an established hematological disease, they may also have other changes associated with medications, metabolism, and nutritional status that must be monitored in laboratory tests.

The complete blood count is the main diagnostic tool in hematology, and it can identify anemia due to nutrient deficiencies, anemia associated with acute or chronic diseases, infections, and leukemias [87].

Fasting glucose is a marker for the detection and monitoring of changes related to serum glucose and diabetes mellitus [88]. In diabetes mellitus, it is also important to monitor glycated hemoglobin for more specific screening, which is considered the best predictor of chronic complications [89].

The lipid profile comprises the biochemical fractions of total cholesterol (TC), high-density lipoprotein (HDL), low-density lipoprotein (LDL), very low-density lipoprotein (VLDL), and triglycerides [90]. It is indicated for evaluating and monitoring patients with known coronary artery disease or other atherosclerotic vascular diseases. It is also requested in patients with associated risk factors, such as systemic arterial hypertension, diabetes mellitus, obesity, and smoking [91, 92].

Total proteins and fractions measure the total amount of albumin and globulin in the blood. It is indicated to diagnose nutritional disorders (such as energy-protein malnutrition), liver disease, and kidney disease [93].

In renal function alterations, uric acid, creatinine and urea values, and other specific tests are generally used as markers. Uric acid is requested for investigation of the risk of cardiovascular diseases and monitoring of nephropathies [94–96]. It should also be requested for obese patients [97], diabetics [98, 99], hypertensive patients [100], and individuals who consume alcohol frequently [101] and requested for monitoring individuals undergoing chemotherapy and radiotherapy [102].

Reticulocytes provide information about the level of erythropoietic activity in the marrow, being used to verify the effectiveness of the treatment of iron deficiency anemia, for the follow-up and monitoring of patients with sickle cell disease [103–105]. In addition, reticulocytes assess bone marrow activity after chemotherapy, radiotherapy, or bone marrow transplantation [105].

The values of lactate dehydrogenase (LDH) are requested in cases where there is suspicion of the occurrence of lesions or cellular damage [106]. Other exams can also be observed since usually in the routine of the multidisciplinary team, they can be available for the nutritionist to view, in either physical or electronic medical records.

In view of this, the nutritionist must describe the exams and indicate those that are outside the normal range and the date of performance.

### 3.8. Semiological Assessment (Physical Examination)

The word semiology derives from the Greek semeion, which means the study of signs. Semiology is the investigation and study of the signs and symptoms presented by the patient, focused on performing the physical examination [107]. Semiology is the chapter of the general pathology of medical science, which aims to teach the correct technique to obtain signs and symptoms of a certain pathogenic state through inspection, palpation, percussion, and auscultation [108]. The practice of semiology is important in inpatient care, where essential issues for the patient's clinical history will be addressed [109].

Semiology or physical examination has become fundamental in the daily care practice of health professionals since it is through anamnesis and a well-developed and structured physical examination that the professional is able to identify signs and symptoms manifested by the patient, associated with their life habits and sociocultural factors that may indirectly or directly influence the health-disease process of that person and, from that, implement the diagnoses and outline effective care lines for the treatment and recovery of the patient [107, 110]. In addition, the use of appropriate communication techniques between professionals, patients, family members, and the community is also encouraged [107].

The semiological nutritional assessment is a clinical method used to detect signs and symptoms associated with protein-calorie malnutrition. These signs and symptoms develop only in the advanced stages of nutritional depletion. Some diseases present symptoms similar to those presented in malnutrition, and it is important to know the patient's clinical history to avoid an incorrect nutritional diagnosis [111, 112]. The changes to look out for are summarized in Table 4.

### 3.9. Anthropometric Assessment

The use of anthropometric indicators is one of the direct parameters of nutritional status and is considered the most appropriate and accessible to be adopted in health services. It has several advantages, of which it is noninvasive, low cost, simple to perform, easy to apply, and standardized. Anthropometric assessment is a method based on the measurement of physical variations in some segments or global body composition [163].

The measurements most used in the anthropometric assessment in the FNC are described as follows: current body weight, height, body mass index, circumferences, and skinfolds. Deeper body composition exams such as dual energy X-ray emission densitometry (DXA) can be seen in the requests of the hematologist, which is very common in the clinical follow-up of children and adults with sickle cell disease [164–167].

In outpatient clinics, it is common that patients who present in special situations require greater care in anthropometric measurements, especially in relation to height [168]. These specific situations, such as wheelchair users, bedridden patients, patients using a prosthesis (due to necrosis of the femoral head—SCD), patients with amputated limbs (due to leg ulcer—SCD), or unevenness of the lower limbs (SCD), can be observed in all patients. It is more common in patients with SCD but can be observed in patients with hemophilia [168].

### 3.10. Assessment of Food Consumption

As in anthropometry, the assessment of food consumption aims to identify nutritional disorders, enabling an appropriate intervention to assist in the recovery or maintenance of the patient's nutritional status.

In clinical practice with HD outpatients, two methods of food inquiry are used in the first consultation: habitual consumption (HC) and anamnesis with food frequency. The method of habitual food consumption (HC), known as food history or dietary history, is a method for evaluating the dietary pattern that consists of an extensive interview with the objective of generating information about the patient's current and past eating habits [169]. Thus, the following must be recorded in the medical record: (1) name, registration, and the date of the collection day; (2) the time, meal, place of the meal, type of food (liquid or solid), type of preparation, household measures, and observations. The record performed must be detailed since this instrument can be used to calculate the macro- and micronutrients of assisted patients.

The Food Frequency Anamnesis method, also called the Food Frequency Questionnaire (FFQ), is considered the most practical and informative retrospective method for evaluating habitual food consumption, providing qualitative information about it [169]. This type of anamnesis can be used in the ward to optimize the release of the patient's diet or in the outpatient clinic when it is not possible to perform an anamnesis by CH. The AA consists of generating information on the frequency of food consumption for a certain period (weekly, monthly, or yearly) [170]. For this, the patient should be asked:

- Number and time of meals/day: write down the number of meals since the patient wakes up as well as the time of each meal.
- Meal locations: note the location where meals are held (work, home, etc.).
- Consumption of milk and dairy products: note whether the milk and yogurt are whole, skimmed, or semiskimmed and the type of cheese (white or yellow). Also, note the frequency and quantity.
- Meat consumption (beef, pork, chicken, fish, offal, and egg): note the quantity and frequency of consumption and the method of preparation.
- Consumption of canned food and sausages (sausages, bologna, salami, and ham): note the type, quantity, and frequency of consumption and the method of preparation.
- Consumption of bread (bread, cookies, and cake): note the type, quantity, and frequency of consumption.
- Consumption of legumes (beans, peas, chickpeas, lentils, etc.): note the amount and frequency of consumption.
- Cereal consumption (rice, pasta, sago, hominy, popcorn, green corn, oats, manioc flour, cornstarch flour, arrowroot flour, rice flour, cornmeal flour, and tapioca flour): note the amount and frequency of consumption and the method of preparation.
- Consumption of fats (butter, margarine, oil, olive oil, mayonnaise, and sour cream): note the amount and frequency of consumption and the method of preparation.
- Consumption of oilseeds (hazelnuts, cashews, Brazil nuts, walnuts, coconut, and peanuts): note the quantity and frequency of consumption and the method of preparation.
- Fruit consumption: write down in numbers the quantity, which fruit, and the frequency.
- Consumption of vegetables: write down in numbers the amount, which vegetable, the frequency, and the type of preparation.
- Water consumption: inform the volume of water consumed. Note: to facilitate your analysis and the patient's understanding, ask him to say in glasses or bottles how much is consumed per day.
- Consumption of teas and/or infused drinks: inform if tea is consumed. Inform which tea is being used and its amount and frequency.
- Consumption of sweets, soft drinks, and chocolates: write down the quantity and frequency in numbers.

As a complement to the assessment of food consumption, the clinical nutritionist can also discuss the time and duration of meals with the patient; the company that prepares and buys the food; the location and frequency of purchase of perishable and nonperishable foods; storage conditions; appetite conditions (important to know if there has been a recent change in appetite, what were the reasons for such change); preference and rejection of food; food intolerance and allergies; food myths and taboos; reasons that led to the change in the eating routine; habits on weekends, holidays, and vacations; and use of nutritional supplements, if the patient has already been on a guided diet or not, and the results (knowing previous experiences, as well as identifying expectations regarding nutritional guidance).

### 3.11. Nutritional Conduct

After all the previous assessments, it is necessary to carry out the nutritional management step. This will depend on the nutrition professional's knowledge and empowerment of hematological diseases [168, 171] to carry out an adequate nutritional approach. In the nutritional care of outpatients, nutritional guidelines are usually offered based on previous nutritional therapeutic planning.

Nutritional management for individuals with hematological disease must be individualized, respecting the specifics of each case and, with the aim of preventing or reversing the decline in nutritional status, as well as preventing progression to severe malnutrition or cachexia, in addition to improving nitrogen balance, reducing proteolysis, and increasing immune response [172, 173].

Nutritional management commonly follows the following directive steps.

#### 3.11.1. Food Plan Prescription

It consists of the nutritional intervention procedure performed through routine instruments for dietary calculation of a detailed and specific food plan in calories, including macronutrients and micronutrients.

For the dietary calculation of a food plan, it is essential to determine the nutritional needs (or energy needs) of the person. Energy needs are defined as the dietary energy intake through food sources of carbohydrates, proteins, and fats, which is necessary to maintain energy balance in healthy people of a defined age, sex, weight, and height, and a compatible degree of physical activity, and in good health [174]. Energy is spent by the human body in different ways, among them, in the form of basal metabolic rate (BMR), which makes up the total energy expenditure (TEE).

To determine the energy needs of people with hematological diseases, health status and physical activity level should be considered, to adjust the nutritional supply of individuals. Thus, energy needs can be obtained through direct and indirect measurements or calculated using formulas that estimate them through the correlation of previously known parameters [175].

The method considered the gold standard for calculating energy expenditure is indirect calorimetry, to guide and determine energy requirements. This method can be used for children and adults; however, it is not routinely used in clinical practice due to its high cost and for requiring a team trained in the handling of a calorimeter [176]. Therefore, energy predictive equations are used, which consider information such as sex, age, weight, height, and physical activity to estimate energy needs [177, 178].

In outpatient clinical practice, some predictive equations can be used to estimate nutritional needs, as this method is quick and easy to perform, does not require a trained evaluator, and is of low cost [179]. There are several predictive equations described in the literature which were developed to be used in healthy or sick individuals. Among the most used equations are FAO/WHO/UNU, Harris–Benedict, Ireton–Jones, and the practical method or “pocket formula,” as it is known [180–183].

It is important to emphasize that the method used to estimate nutritional needs will depend on several factors, such as the underlying hematologic disease, since, depending on the method chosen, it may overestimate or underestimate the needs, which may lead to damage to the nutritional status of the individual [176, 179].

Daily macronutrient and micronutrient requirements can be calculated according to the dietary reference intakes, estimated according to sex and age group [174, 184]. Throughout the course of the hematologic disease, patients may require greater macronutrient and micronutrient requirements. The nutritionist must be aware of the *Tolerable Upper Intake Level* to not exceed the upper limit since the values above this are within the competence of the physician and/or pharmacist. From this, the food plan to be prescribed must also include nutritional guidelines based on the biological and sociocultural aspects of the assisted individual.

#### 3.11.2. Nutritional Guidelines

This step can be defined as the procedure in which the nutritional intervention and dietary prescription are carried out through nutritional counseling and individual meal plan planning and offer/guidance of educational materials (Nutritional Guidelines—ONs and Homemade Recipes—ERs) to assist patients. These nutritional guidelines may be specific for hematological diseases or linked to other health conditions such as diabetes and hypertension [185]. In the medical record, the instructions to the patient regarding consumption/eating habits, water intake, number of meals, etc., should be briefly recorded [185].

In Brazil, nutritionists use the Food Guide for the Brazilian Population as a basis for nutritional guidelines, which is an official document that addresses the principles and recommendations of an adequate and healthy diet for the Brazilian population, configuring itself as a support instrument to food and nutrition education actions [186].

Nutritional education guides are educational tools that improve the population's eating patterns, contribute to health promotion, and as information to promote education for the adoption of a more adequate diet by all people and that consider the sociocultural aspects of the population [186].

#### 3.11.3. Nutritional Therapy Planning for Upcoming Consultations

This stage comprises the therapeutic planning of nutritional treatment in the short, medium, and long term, including treatment adherence, progression of weight gain/loss, dietary adequacy, and reassessment of the treatment employed by the clinical nutritionist. In the medical record, topics to be addressed in the next appointment must be recorded, and if necessary, perform any anthropometric measurements and address pending issues/guidelines [12].

The qualification and specialization of nutrition in hematology should not be focused only on the general nutritional aspects of patients with HD. Nutritional management depends on several factors, ranging from the technical capacity of the clinical nutritionist, as well as whether their technical-scientific knowledge is sufficient for dialog with other specialists, that is, how much the nutritionist knows of the physiology of hematology as well as the pathophysiology of hematological diseases and, therefore, carry out a better intervention from a nutritional point of view [12].

Therefore, the outpatient follow-up of these patients requires specialization by the clinical nutritionist, especially regarding questions related to the investigation of nutritional aspects and eating habits that are directly or indirectly related to the individual's underlying disease [168, 171].

**Figure 1:**
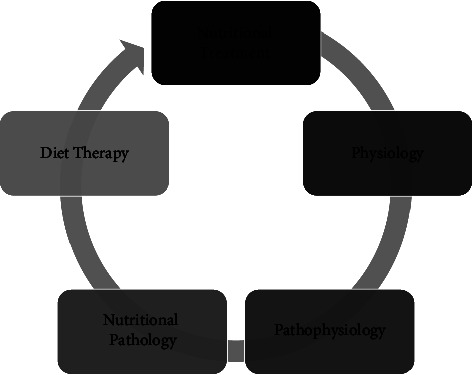
Steps of qualification and specialization of nutrition in hematology.

The traditional clinical nutrition services of reference centers in hematology must bear in mind that it is necessary to reframe nutritional care in the light of scientific knowledge in hematology and in the practice of evidence-based nutritional care, to propose a more effective and assertive nutritional treatment shown in Figure 1 [168, 171].

#### 3.11.4. Subsequent Query

For longitudinal and continuous follow-up of patients, as well as to assess adherence to the short-, medium-, and long-term treatment plan, it is necessary to schedule subsequent appointments, also called follow-up appointments, where adherence, progression, dietary adequacy, and reassessment are performed, and discharge from treatment [12].

The first return called adherence return comprises the moment in which the patient is encouraged to report how it was the period in which he had to follow the prescribed nutritional guidelines and how much he dedicated himself to practicing them (with a report of what were the facilities and difficulties to carry out them). For this, the nutritionist must have a complete copy of the record (xerox or on the computer) including what was prescribed to the patient.

At first, the nutritionist must extract from the patient a self-assessment of everything that was agreed upon in the first consultation. This simple conduct is important in the process of both adherence and stimulation of patients' self-care [12]. This conduct is also important in creating a bond, especially with more severe patients or those dependent on family support [12]. As an example, the patient can be asked what grade he would give himself (0 to 10) in relation to the guidelines he received and managed to practice after the first consultation, and based on the answer given, the nutritionist could question the reason for this grade and let the patient report their difficulties and successes [12, 168].

This proposal allows the nutritionist to measure how much the patient adhered to the conduct performed in the first consultation and in a second moment, each step prescribed by the nutritionist could be questioned again by the patient, to sediment the information in the first consultation. This is done to ensure adherence to nutritional treatment [12, 168].

## 4. Conclusion and Recommendation

To plan, direct, and implement health, food, and nutrition actions in hematology reference centers, it is necessary to know the reality, dynamics, and risks that the population with hematological disease is inserted into.

In addition to knowledge and specialization in hematological diseases, the clinical nutritionist, within the scope of his/her competencies, must have an expanded look at the health needs of their patients, incorporating collective health knowledge and practices in the outpatient and/or hospital context.

The first nutrition consultation in adults with hematological disease represents investigative steps that help the clinical nutritionist in nutritional management, allowing a longitudinal nutritional therapeutic planning of hematological patients assisted in the outpatient clinic.

We hope with this review to have filled a gap in the field of hematology, being a guide not only for nutritionists but also for hematologists and other members of the multidisciplinary team in the outpatient and hospital environment.

## Figures and Tables

**Table 1:** Types of hematological diseases commonly referred to the clinical nutrition service at a reference center in Brazil.

Proliferatives and infiltratives	Indolent	Prolymphocytic chronic lymphocytic leukemia
		Chronic myeloid leukemia
		Lymphomas
		Multiple myeloma
		Low-risk myelodysplastic syndrome
		CMS: myelofibrosis: primary or terminal phase
	Aggressive	ALL, AML
		High-risk myelodysplastic syndrome
	Lysosomal storage disease	Gaucher disease (type 1, type 3)
		Niemann–Pick disease

Anemias	Aplastic	PNH
	Hemolytic	Acquired: AHA
		Hereditary: G6PD deficiency, spherocytosis
		Hemoglobinopathies: thalassemia and sickle cell disease

Changes in coagulation	Von Willebrand disease	Quantitative (type I; type 1C; type 3; low)
		Qualitative (type 2A; type 2B; type 2M; type 2N)
	Hemophilia	Type A (severe, moderate, mild)
		Type B (severe, moderate, mild)
	Thrombocytopenia	ITP
		Autoimmune disease
		TTP
	Others	Acquired disability
		Factor V deficiency
		Factor VII deficiency

Source: adapted from WHO, 2004 [4]; Knust et al., 2016 [5]; Ng and Di Paola, 2018 [6]; Blanchete et al., 2014 [7]. CMS, chronic myeloproliferative syndrome; ALL, acute lymphoid leukemia; ALM, acute myeloid leukemia; PNH, paroxysmal nocturnal hemoglobinuria; AHA, autoimmune hemolytic anemia; G6PD, glucose-6-phosphate dehydrogenase; type 1C, type 1 clearance; ITP, idiopathic thrombocytopenic purpura; TTP, thrombotic thrombocytopenic purpura.

**Table 2:** Important questions for the first nutritional consultation.

Allergies and/or food intolerances		
Signs and symptoms when eating a certain food	If positive	If negative
Allergy: spots and/or itchy skin, dermatitis, edema (face, glottis, etc.) Intolerance: diarrhea, nausea, vomiting, and abdominal distension	Record signs and symptoms. Questioning the type, quantity, and frequency of food eaten	Recording in medical records denies food allergies and/or food intolerances
Picamalacia		
Current or past period	If positive	If negative

Ingest inedible substances such as: brick, toothpaste, soap, chalk, ice in the refrigerator, paper, rubber, earth, toilet paper and etc	Question and register the type, frequency, and quantity of the substance ingested; if ingestion was in the past, record the age group, period, or related events such as pregnancy, school, psychological changes, or family problems	Record which patient denies picamalacia currently and in the past

Socioeconomic and lifestyle information		
Question the patient	If positive	If negative

Exercise or physical activity, alcohol consumption, and illicit drug use	Record the type, frequency, and period	Recording in the medical record denies the practice of exercise or activity, consumption of alcohol, drugs, and smoking (current or in the past)
Smoking	Register: how many cigarettes and/or packs, frequency period/time: current or past for former smokers	
Socioeconomic status	Record the level of education (no education, elementary school, high school, and higher education); whether you are working and whether or not you have an employment relationship; monthly per capita household or individual income (minimum wages); housing situation (lives alone or with other people); marital status (single, married, divorced, and widowed); regular access to food (register if you can buy food in sufficient quantity and quality, without compromising other basic needs)	If they are not included in social programs and experience situations that could generate a state of social and food insecurity, the unit's social service and the social support networks must be activated
Receive some type of benefit or income transfer program	Register which benefits or income transfer program is included such as family allowance/Auxílio Brasil, BPC, or retirement or pension	

BPC, benefit of continued installment.

**Table 3:** Important questions regarding changes in the digestive tract in the first nutritional consultation.

Digestive tract alterations	Definition	Scientific references
Dysphagia	Symptom of swallowing dysfunction that occurs between the mouth and the stomach	[50]
Odynophagia	Painful swallowing symptom	[51]
Stomatitis	Inflammatory disease of the oral mucosa, often accompanied by pain	[52]
Dysgeusia	Taste disorder characterized by a distortion in taste perception. Total dysgeusia is defined as the inability to interpret all the basic tastes, which is most often associated with mineral deficiency. This condition can cause loss of appetite and even malnutrition	[53]
		[54]
Xerostomia	Dry mouth condition, altered taste, and/or reduced salivary flow, which limit the quality of life in terms of poor oral hygiene, halitosis, and difficulty speaking, chewing, and/or swallowing	[55]
Ageusia	Loss of taste	[56]
Anorexia	Loss of appetite for food, which can be caused by various physiological and pathophysiological conditions	[57]
Nausea	Vague and unpleasant feeling of being sick with the feeling that vomiting may occur. Nausea, a subjective symptom, is often preceded by feelings of anorexia and is often accompanied by objective symptoms of pallor, hypersalivation, diaphoresis, and tachycardia	[58]
Vomiting	Characterized by forced ejection of gastric contents through the mouth	[58]
Diarrhea	Disorder of the bowel with increased frequency of defecation or increased stool weight	[59]
Abdominal pain	Gastrointestinal disorder with acute or chronic expression and with different etiologies	[60]
Dyspepsia	Presence of one or more of the following four symptoms for three months in the first six months of symptom onset: uncomfortable postprandial fullness, uncomfortable early satiety, uncomfortable epigastric pain, and uncomfortable epigastric burning	[61]
Heartburn	Discomfort or retrosternal burning sensation, which radiates to the epigastric and neck	[62]
Postprandial fullness	Symptom associated with delayed gastric emptying, mainly due to compromised antral function	[63]
Constipation	Disorder of the gastrointestinal tract, which can result in infrequent stools, difficult passage of stools with pain and stiffness	[64]

**Table 4:** Physical manifestations associated with malnutrition and nutritional deficiencies.

Signs and symptoms		Deficiency	Scientific references
Mouth	Angular stomatitis, cheilosis	B2, B3, B6	[113, 114]
	Bleeding gums	Vitamin C, B2	[115, 116]

Hair	Loss of natural, dry shine; thin and sparse; depigmented; flag sign; easy to start without pain	Kwashiorkor and marasmus	[117–119]

Tongue	Inflamed tongue/glossitis/geographic tongue	B3, B5, B6, B9, B12, iron, zinc	[120–123]
	Magenta tongue (purple)	B2	[124]
	Fissure in the tongue	B3	[120]
	Atrophy of the lingual papillae	B2, B3, iron	[125, 126]
	Reduced taste sensitivity	Zinc	[127]

Glands	Thyroid enlargement/goiter	Iodine	[128]
	Increased parathyroid	Starvation	[129]

Skin	Xerosis, follicular hyperkeratosis	Vitamin A	[130, 131]
	Petechiae (small hemorrhages), purpura, dermatoporosis	Vitamin C	[132]
	Hyperpigmentation/rosacea	B3	[133]
	Pallor	Iron, B12, B9	[134]
	Nasolabial seborrhea/seborrheic dermatitis	B2, essential fatty acids	[135, 136]
	Vulvar and scrotal dermatosis	B2	[137]
	Desquamative cosmetic dermatosis	Kwashiorkor	[138]
	Pellagra	B3	[139]
	Hurts easily/bruising	Vitamin C or K	[140, 141]

Nails	Brittle, rough; koilonychia	Iron	[142, 143]

ST	Edema	Kwashiorkor	[144, 145]

Chest	Respiratory muscle weakness/diaphragmatic dysfunction	Protein	[146]

GS	Hepatosplenomegaly	Kwashiorkor	[147]

Musculoskeletal system	Epiphyseal widening, persistence of the opening of the anterior fontanelle and X-shaped leg	Vitamin D	[148]
	Rachitic rosary	Vitamin D	[149]

NS	Psychomotor alteration	Kwashiorkor	[150]
	Loss of sense of vibration, position and ability to contract the wrist, motor weakness, paraesthesia	B1, B12	[151, 152]
	Insanity	B1, B3, B12	[153–155]
	Peripheral neuropathy	B1, B6, vitamin E	[156, 157]

CS	Heart enlargement, tachycardia	B1	[158, 159]

Source: adapted from [112, 160–162]. ST, subcutaneous tissue; GS, gastrointestinal system; NS, nervous system; CS, cardiovascular system. B-complex vitamins: B1, thiamine; B2, riboflavin; B3, niacin; B5, pantothenic acid; B6, pyridoxine; B9, folic acid; B12, cyanocobalamin.

## Data Availability

The data used to support the findings of this study can be obtained from the corresponding author upon request.
